# Allergen skin test reactivity and asthma are inversely associated with ratios of IgG4/IgE and total IgE/allergen‐specific IgE in Ugandan communities

**DOI:** 10.1111/cea.13834

**Published:** 2021-02-05

**Authors:** Gyaviira Nkurunungi, Jacent Nassuuna, Harriet Mpairwe, Joyce Kabagenyi, Margaret Nampijja, Richard E. Sanya, Emily L. Webb, Alison M. Elliott

**Affiliations:** ^1^ Immunomodulation and Vaccines Programme Medical Research Council / Uganda Virus Research Institute and London School of Hygiene and Tropical Medicine (MRC/UVRI and LSHTM) Uganda Research Unit Entebbe Uganda; ^2^ Department of Non‐Communicable Disease Epidemiology London School of Hygiene and Tropical Medicine London UK; ^3^ College of Health Sciences Makerere University Kampala Uganda; ^4^ MRC Tropical Epidemiology Group, Department of Infectious Disease Epidemiology London School of Hygiene and Tropical Medicine London UK; ^5^ Department of Clinical Research London School of Hygiene and Tropical Medicine London UK; ^6^ Present address: Centre for Geographic Medicine Research‐Coast Kenya Medical Research Institute Kilifi Kenya; ^7^ Present address: Maternal and Child Wellbeing Unit African Population and Health Research Centre Nairobi Kenya

**Keywords:** allergen, asthma, IgE, IgG4, *Schistosoma*, skin prick test

## Abstract

**Background:**

Serum inhibition of allergen‐specific IgE has been associated with competing IgG4 and non‐specific polyclonal IgE. In allergen immunotherapy, beneficial responses have been associated with high IgG4/IgE ratios. Helminths potentiate antibody class switching to IgG4 and stimulate polyclonal IgE synthesis; therefore, we hypothesized a role for helminth‐associated IgG4 and total IgE in protection against atopic sensitization and clinical allergy (asthma) in tropical low‐income countries.

**Methods:**

Among community residents of Ugandan rural *Schistosoma mansoni* (*Sm*)–endemic islands and a mainland urban setting with lower helminth exposure, and among urban asthmatic schoolchildren and non‐asthmatic controls, we measured total, *Schistosoma* adult worm antigen (SWA)–specific, *Schistosoma* egg antigen (SEA)–specific and allergen (house dust mite [HDM] and German cockroach)–specific IgE and IgG4 by ImmunoCAP^®^ and/or ELISA. We assessed associations between these antibody profiles and current *Sm* infection, the rural‐urban environment, HDM and cockroach skin prick test (SPT) reactivity, and asthma.

**Results:**

Total IgE, total IgG4 and SWA‐, SEA‐ and allergen‐specific IgE and IgG4 levels were significantly higher in the rural, compared to the urban setting. In both community settings, both *Sm* infection and SPT reactivity were positively associated with allergen‐specific and total IgE responses. SPT reactivity was inversely associated with *Schistosoma*‐specific IgG4, allergen‐specific IgG4/IgE ratios and total IgE/allergen‐specific IgE ratios. Asthmatic schoolchildren, compared with non‐asthmatic controls, had significantly higher levels of total and allergen‐specific IgE, but lower ratios of allergen‐specific IgG4/IgE and total IgE/allergen‐specific IgE.

**Conclusions and clinical relevance:**

Our immuno‐epidemiological data support the hypothesis that the IgG4–IgE balance and the total IgE–allergen‐specific IgE balance are more important than absolute total, helminth‐ or allergen‐specific antibody levels in inhibition of allergies in the tropics.

## 1 INTRODUCTION

Several immuno‐epidemiological studies have shown that helminth infections are associated with protection against allergy‐related conditions,^1^, ^2^, ^3^, ^4^, ^5^, ^6^, ^7^, ^8^, ^9^ and highlighted the potential role of helminths in rural‐urban differences in prevalence of allergy‐related diseases in the tropics.^10^, ^11^ We have previously shown that maternal hookworm infection modifies risk factors for childhood eczema, implying that early‐life exposure to helminths may also establish protection against allergy‐related diseases.^12^ Experimental human and animal studies have demonstrated that the mechanisms through which helminths may down‐modulate allergic responses are extensive,^13^, ^14^ covering almost the entire range of the allergy‐related immunological pathway. Antibody‐mediated immune mechanisms of protection are less understood compared with cell‐mediated mechanisms: current theories involve helminth‐induced immunoglobulin (Ig) G4 and polyclonally stimulated IgE.

By inducing high levels of interleukin (IL)‐10, helminths can promote immunoglobulin class switching to IgG4. Moreover, chronic helminth infection is associated with elevated serum IgG4 levels,^15^ and serum inhibition of helminth‐specific IgE has been associated with competing IgG4 in a *Schistosoma mansoni* (*Sm*)–endemic setting.^16^ Several human studies have suggested that helminth‐induced IgG4 is important in protection against allergy: among school‐age children in rural Ecuador, *Ascaris*‐specific IgG4 was inversely associated with allergen skin prick test (SPT) reactivity^2^; in a *Sm*‐endemic Ugandan rural setting, we observed an inverse association between house dust mite–specific IgG4/IgE ratios and reported recent wheeze.^17^

Studies conducted four decades ago^18^, ^19^ provided initial evidence that parasitic helminths mediate production of high levels of IgE that is not specific to the parasite, or to inhalant allergens. Non‐specific polyclonally stimulated IgE has been proposed to inhibit allergic responses by competing with allergen‐specific IgE to saturate IgE receptors,^20^ reducing the chances that an allergen will result in cross‐linking of FcεRI‐bound IgE and hence effector cell degranulation.^14^, ^21^ However, there is little evidence for polyclonal IgE‐mediated protection against allergic inflammation, and high IgE titres have previously been linked to increased expression of IgE receptors on human basophils,^22^ signifying potential for polyclonally stimulated IgE in increased effector cell degranulation. Therefore, the question of whether polyclonal IgE mitigates allergic responses remains unresolved.

Large, well‐defined immuno‐epidemiological studies in helminth‐endemic settings are required to better understand population‐level interactions between allergy‐related disease and helminth‐ and allergen‐associated IgG4 and polyclonal IgE profiles. This will contribute to bridging the gap between understanding basic antibody mechanisms and clinical applications. We used the opportunity presented by studies designed to assess the epidemiology of allergy‐related disease in (1) *Sm*‐endemic Ugandan rural fishing villages,^17^, ^23^, ^24^, ^25^ (2) proximate urban communities with lower helminth exposure^11^ and (3) urban asthmatic schoolchildren and non‐asthmatic controls.^26^, ^27^ Samples collected enabled us to measure total IgE (as a proxy for polyclonally stimulated IgE), total IgG4 and *Sm*‐ and allergen‐specific IgE and IgG4 profiles, and to analyse their associations with current *Sm* infection, the rural‐urban environment, allergic sensitization and asthma.

## 2 METHODS

### 2.1 Study design and population

The current investigation was conducted using samples from participants of cross‐sectional community surveys in rural^24^ and urban^11^ Uganda, and from a case‐control study investigating asthma risk factors among 5‐ to 17‐year‐old schoolchildren in urban Uganda.^26^

The rural survey was the three‐year outcome survey (September 2015–August 2016) of the Lake Victoria Island Intervention Study on Worms and Allergy‐related diseases (LaVIISWA; ISRCTN47196031).^23^, ^24^, ^25^ The LaVIISWA trial was conducted in 26 *Sm*‐endemic rural fishing villages of Koome islands, Lake Victoria. It was an open cluster‐randomized trial of community‐wide standard versus intensive anthelminthic mass drug administration (MDA).

The urban survey (September 2016–September 2017) was designed to collect data for comparison with the rural survey.^11^ It was conducted in Entebbe municipality, a lower helminth exposure, urban setting situated on the northern shores of Lake Victoria (approximately 35 km from Koome islands and 40 km from Uganda's capital, Kampala).^11^ Procedures in the urban survey mirrored those in the rural survey; however, urban survey participants were not randomized to standard versus intensive MDA.

The asthma case‐control study (May 2015–July 2017)^26^ enrolled children with doctor‐diagnosed asthma (“cases”) and non‐asthmatic controls from primary and secondary schools in Entebbe municipality and Katabi sub‐county in Wakiso, Uganda. The International Study on Allergy and Asthma in Children (ISAAC) questionnaire^28^ was used for screening, to identify participants who reported wheezing in the previous 12 months. These children then underwent a comprehensive clinical evaluation of medical and treatment history, and examination for asthma signs and symptoms by study clinicians in order to make an asthma diagnosis. Lung function tests and asthma control tests^26^, ^27^ were conducted to assess asthma control. As reported elsewhere,^27^ only three cases had abnormal lung function tests, 85% had well‐ or partly controlled asthma and 15% had poorly controlled asthma. All cases were seen at one time‐point, started on recommended treatment and referred for further management; therefore, asthma severity or response to treatment could not be assessed.^27^ Non‐asthmatic controls were children in the same class as cases, with no history of wheezing or any asthma symptoms, randomly selected using a Stata program (StataCorp, College Station, TX, USA) to obtain a control/case ratio of 2:1.

### 2.2 Parasitological examinations

In all studies, we used the stool Kato‐Katz (KK) technique^29^ for diagnosis of *Sm*, hookworm (*Necator americanus*), *Ascaris lumbricoides* and *Trichuris trichiura* infections. For each participant, one stool sample (prepared on two slides) was examined under a microscope by two laboratory technologists blinded to each other's result. In the rural and urban surveys, stool was further suspended in 70% ethanol, stored at −80°C and later examined for *Sm*, *Strongyloides stercoralis* and hookworm infections using multiplex real‐time PCR.^30^, ^31^ Infection with *Schistosoma haematobium* has not been documented in our study areas.^32^ Plasma samples were assessed for *Schistosoma* adult worm antigen (SWA)‐ and *Schistosoma* egg antigen (SEA)–specific IgE and IgG4 by ELISA (details in Appendix S1).

### 2.3 Assessment of allergy‐related outcomes

Wheezing in the previous 12 months was also assessed in the rural and urban surveys using interviewer‐administered standardized paper and video ISAAC questionnaires. Skin prick test (SPT) reactivity to crude extracts from *Dermatophagoides* mix (*D*.* pteronyssinus and D*. *farinae*), *Blomia tropicalis* and *Blattella germanica* (ALK‐Abelló; supplied by Laboratory Specialities [Pty] Ltd., SA) was assessed using standard methods.^33^ These allergens are common in the study settings.^34^ We used an in‐house ELISA to quantify crude house dust mite (*D*.* pteronyssinus*, HDM) and German cockroach (*B*.* germanica*) extract–specific plasma IgG4 and total IgG4. Total IgE and allergen extract–specific IgE were measured using the ImmunoCAP^®^ IgE test (Thermo Fisher Scientific, Uppsala, Sweden).^35^ Allergen extract–specific IgE was additionally measured using an in‐house ELISA, to facilitate determination of IgG4/IgE ratios, since, for logistical reasons, we could not measure allergen‐specific IgG4 by ImmunoCAP^®^. The in‐house assays are described in this article's supplementary information Appendix S1.

### 2.4 Statistical methods

Statistical analyses were conducted using Stata 15.0 (StataCorp). Graphs were drawn using GraphPad Prism (version 8.2.1; Fay Avenue, CA, USA). We accounted for study design in all analyses involving the rural and urban community surveys: Stata “svy” commands were used to allow for the non–self‐weighting clustering by village in the rural survey and for clustering by sub‐ward in the urban survey.^11^ Initial analyses in the rural survey assessed the impact of intensive versus standard MDA on IgE and IgG4 profiles. For this, we used a cluster‐level approach^36^ (previously described in other analyses for this study^24^) that involved comparing cluster‐specific means of antibody concentrations between trial arms, with 95% confidence intervals and p‐values calculated using the t distribution. For all subsequent analyses, standard and intensive trial arm participants in the rural survey were grouped together.

Next, participant characteristics were tabulated and compared between urban and rural settings separately, between asthma cases and controls, using logistic and linear regression for categorical and continuous variables, respectively.

Independently for each of the three studies, we conducted crude and age‐ and sex‐adjusted cross‐sectional analyses to investigate the direction and the strength of association between antibody concentrations or ratios (total, *Schistosoma*‐specific and allergen‐specific IgE and IgG4 concentrations; allergen‐specific IgG4/IgE, total IgE/allergen‐specific IgE, total IgG4/total IgE ratios) and *Sm* infection or SPT reactivity. In the asthma case‐control study, we additionally investigated associations between asthma and antibody concentrations and ratios. Rural‐urban comparisons of antibody profiles were conducted using data from the rural and urban surveys. Most raw antibody concentrations and ratios were skewed, so for all analyses, linear regression models of log‐transformed data were used. ImmunoCAP^®^ allergen–specific IgE concentrations were log_10_ (+0.001)‐transformed, while all other antibody data were log_10_ (+1)‐transformed. The results were then back‐transformed to obtain geometric mean ratios (GMRs) and 95% confidence intervals (CIs).

### 2.5 Ethics statement

Our studies were approved by research ethics committees of Uganda Virus Research Institute (reference numbers: GC/127/12/05/03, GC/127/16/02/547 and GC/127/14/09/481) and London School of Hygiene and Tropical Medicine (reference numbers: 6187 and 10709), and the Uganda National Council for Science and Technology (reference numbers: HS1183, HS2036 and HS1707). We obtained written informed consent from all participants and/or their legal guardians. Informed assent was obtained from children ≥8 years.

## 3 RESULTS

### 3.1 Characteristics of study participants

**FIGURE 1:**
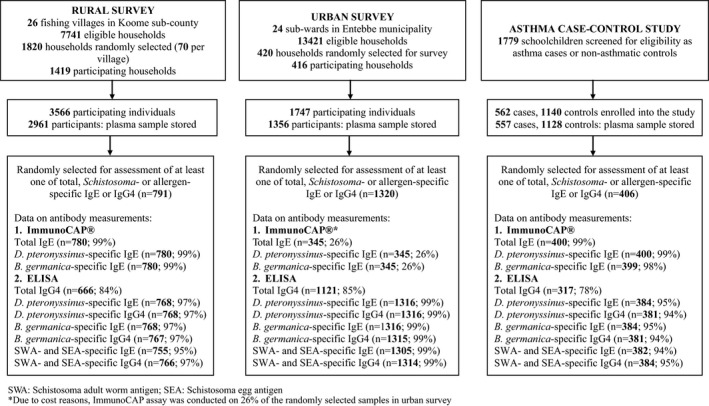
Study flow chart

Flow charts of the three studies are shown in Figure 1. Plasma samples were available for 2961, 1356 and 1685 participants of the rural survey, urban survey and asthma case‐control study, respectively. Antibody measurements were conducted in a subset of randomly selected samples per study: 791, 1320 and 406 plasma samples from the rural survey, urban survey and asthma case‐control study, respectively, were included in assessment of at least one of total, *Schistosoma*‐ or allergen‐specific IgE or IgG4. There was no effect of intensive versus standard anthelminthic MDA on antibody profiles (Table S1), hence data from both trial arms in the rural survey were combined for all subsequent analyses.

Table 1 shows characteristics of participants for whom we obtained data on total, *Schistosoma*‐ or allergen‐specific IgE or IgG4. Participant characteristics were compared between the rural and urban surveys, and between asthma cases and controls. We have previously reported related comparisons in these studies, albeit in somewhat different subsets of participants.^37^ Rural participants, compared with urban participants, were more likely to be older (*p* = .001) and male (*p* = 0.003). Prevalence of SPT reactivity to *Dermatophagoides* mix and *B*.* tropicalis* was higher among urban participants (*p* = .001). However, rural participants had higher median total IgE levels (*p* < .001), and higher prevalence of IgE sensitization to crude German cockroach (ImmunoCAP^®^ concentration ≥0.35 kU/L) [*p* = .001], ELISA‐detectable cockroach‐specific IgE (≥312.5 ng/ml) [*p* = .001], and infection with *Sm* (*p* < .001) and at least one nematode (*p* < .001). Reported wheeze was rare in both settings.

**TABLE 1:** Characteristics of study participants

Characteristics	Rural survey	Urban survey	*p* ^b^	Case‐control study on asthma in schoolchildren n/N (%)		*p*
	*n/N* (%)^a^	*n/N* (%)^a^		Controls	Asthma cases	
Socio‐demographic						
Age in years, median (IQR)	**28 (21, 36)**	21 (9, 32)	**.001**	10 (8, 13)	**11 (10, 14)**	**.023**
Male sex	**382/788 (46.4)**	510/1315 (38.8)	**.003**	93/200 (46.7)	92/200 (46.0)	.847
Allergy‐related outcomes						
Skin prick test reactivity						
*Dermatophagoides* mix	89/788 (10.2)	**223/1270 (17.6)**	**.001**	49/200 (24.5)	**91/198 (45.9)**	**<.001**
*Blomia tropicalis*	55/788 (6.9)	**180/1270 (14.2)**	**.001**	46/200 (23.0)	**90/198 (45.4)**	**<.001**
*Blattella germanica*	100/787 (14.1)	182/1273 (14.3)	.913	33/200 (16.5)	48/198 (24.2)	.056
asIgE sensitization (≥0.35 kU/L, ImmunoCAP)						
*D. pteronyssinus*	264/780 (33.2)	104/345 (30.1)	.421	72/200 (36.0)	**117/200 (58.5)**	**<0.001**
*Blattella germanica*	**393/780 (49.8)**	118/345 (34.2)	**<.001**	90/200 (45.0)	**112/199 (56.3)**	**0.025**
Detectable asIgE (≥312.5 ng/ml, ELISA)^c^						
*D. pteronyssinus*	312/766 (39.9)	496/1313 (37.8)	.452	71/190 (37.4)	**105/191 (54.0)**	**.001**
*Blattella germanica*	**349/766 (45.4)**	492/1313 (37.5)	**.001**	69/190 (36.3)	**96/191 (50.3)**	**.006**
Total IgE (kU/L, ImmunoCAP), median (IQR)	**672 (250, 1942)**	159 (57, 523)	**<.001**	279 (98, 648)	**487 (115, 1248)**	**.018**
Wheeze in last 12 months	25/781 (2.8)	24/1157 (2.1)	.377	0/200 (0.0)	200/200 (100.0)	
Helminth infections						
*S*.* mansoni* (KK)	**188/686 (29.4)**	79/1079 (7.3)	**<.001**	8/196 (4.1)	12/184 (6.5)	0.291
*S*. *mansoni* intensity (KK)						
Uninfected	498/686 (70.6)	**1000/1079 (92.7)**		188/196 (95.9)	172/184 (93.5)	
Low	**95/686 (14.7)**	38/1079 (3.5)		4/196 (2.0)	7/184 (3.8)	
Moderate	**53/686 (8.9)**	27/1079 (2.5)		3/196 (1.5)	3/184 (1.6)	
Heavy	**40/686 (5.8)**	14/1079 (1.3)	**<.001**	1/196 (0.5)	2/184 (1.1)	.272
*S*.* mansoni* (PCR)^d^	**313/686 (47.4)**	190/1073 (17.7)	**<.001**			
Any nematode^e^	**160/688 (21.2)**	110/1086 (10.1)	**<.001**	14/202 (6.9)	22/201 (10.9)	.161

Table shows characteristics for individuals with data on total, *Schistosoma*‐ or allergen‐specific IgE and IgG4. *p*‐values are shown for differences in characteristics between rural and urban survey participants and between asthmatic schoolchildren and non‐asthmatic controls.

Abbreviations: asIgE, allergen‐specific IgE; CCA, circulating cathodic antigen; IQR, interquartile range; KK, Kato‐Katz; PCR, polymerase chain reaction; SEA, *Schistosoma* egg antigen; SWA, *Schistosoma* adult worm antigen.

^a^ Percentages were adjusted for survey design. Percentages / medians that were significantly higher in one group compared with the other (*p* ≤ .05) are highlighted in bold. Adjusting for age and sex had little impact on these differences.

^b^ *p*‐values obtained from survey design‐based logistic or linear regression.

^c^ Lower detection limit was 15.625 ng/ml. 20‐fold diluted plasma samples were used; hence, the lower detection limit in undiluted plasma was calculated as 312.5 ng/ml.

^d^ Information not collected in the asthma case‐control study.

^e^ Rural survey and urban survey: infection with any of *A*. *lumbricoides* (KK), *N*. *americanus* (PCR), *T*. *trichiura* (KK) or *S*. *stercoralis* (PCR). Asthma case‐control study: infection with any of *A. lumbricoides* (KK), *N*. *americanus* (KK) or *T*. *trichiura* (KK). Data on S. stercoralis infection not collected in the asthma case‐control study.

Compared to non‐asthmatic controls, asthma cases were on average older, and more likely to be SPT positive, and to have higher total and allergen‐specific IgE levels. Helminth infection prevalence was low in the asthma case‐control study and comparable between cases and controls (Table 1).

### 3.2 Associations between antibody (IgE and IgG4) concentrations and current *S. mansoni* infection and allergen skin prick test reactivity

In the rural survey (Table 2), *Sm* infection was positively associated with total and SWA‐ and SEA‐specific IgE and IgG4 (*p* < .001), and cockroach‐specific IgG4 concentrations (*p* = .002). Cockroach SPT reactivity was positively associated with ImmunoCAP^®^‐determined cockroach‐specific IgE (adjusted GMR [95% CI]: 8.30 [5.16, 13.35], *p* < .001) and total IgE concentrations (1.70 [1.21, 2.39], *p* = .003). *Dermatophagoides* (hereinafter HDM) SPT reactivity was positively associated with HDM‐specific IgE (ImmunoCAP^®^: 30.7 [17.43, 54.25], *p* < .001; ELISA: 8.47 [3.52, 20.40], *p* < .001), but inversely associated with SWA‐specific IgG4 (0.48 [0.27, 0.86], *p* = .016).

**TABLE 2:** Rural survey: associations between antibody (IgE and IgG4) concentrations and current *S*.* mansoni* infection and skin prick test reactivity

Antigen	Antibody	Geometric mean		aGMR (95% CI)^d^, ^e^	*p* value
		*Sm*−^a^	*Sm+*		
SWA	IgE^c^	**3806**	**5263**	**1.33 (1.19, 1.49)**	**<.001**
	IgG4^c^	**43,417**	**147,139**	**2.98 (1.87, 4.74)**	**<.001**
SEA	IgE^c^	**3645**	**5206**	**1.43 (1.25, 1.64)**	**<.001**
	IgG4^c^	**31,284**	**256,465**	**6.13 (2.84, 13.24)**	**<.001**
House dust mite	IgE^b^	0.13	0.18	1.34 (0.85, 2.12)	.193
	IgE^c^	24.25	27.16	1.29 (0.78, 2.11)	.305
	IgG4^c^	11.31	14.26	1.35 (0.98, 1.85)	.065
German cockroach	IgE^b^	0.31	0.33	0.93 (0.58, 1.49)	.748
	IgE^c^	30.79	37.04	1.07 (0.55, 2.08)	.848
	IgG4^c^	**8.73**	**21.43**	**1.98 (1.12, 3.49)**	**.020**
	Total IgE^b^	**487.06**	**983.74**	**1.75 (1.37, 2.23)**	**<.001**
	Total IgG4^c^	**14,015.76**	**29,461.26**	**2.30 (1.47, 3.60)**	**.001**

		*Cockroach SPT‐* ^a^	*Cockroach SPT+*		
SWA	IgE^c^	4481	4316	1.04 (0.86, 1.27)	.671
	IgG4^c^	86,035	58,160	0.87 (0.37, 2.01)	.727
SEA	IgE^c^	4348	4327	0.99 (0.88, 1.14)	.990
	IgG4^c^	90,368	93,691	1.22 (0.49, 2.99)	.655
German cockroach	IgE^b^	**0.241**	**1.982**	**8.30 (5.16, 13.35)**	**<.001**
	IgE^c^	32.87	73.13	1.33 (0.46, 3.87)	.584
	IgG4^c^	13.31	19.16	1.26 (0.61, 2.60)	.516
	Total IgE^b^	**652.99**	**934.25**	**1.70 (1.21, 2.39)**	**.003**
	Total IgG4^c^	18,804.52	27,183.94	1.27 (0.74, 2.19)	.371

		*Dust mite SPT‐* ^a^	*Dust mite SPT* ^a^		
SWA	IgE^c^	4520	3969	0.94 (0.83, 1.08)	.383
	IgG4^c^	**90,655**	**36,677**	**0.48 (0.27, 0.86)**	**.016**
SEA	IgE^c^	4389	4005	0.97 (0.85, 1.11)	.623
	IgG4^c^	89,975	96,456	1.40 (0.72, 2.73)	.309
House dust mite	IgE^b^	**0.10**	**2.27**	**30.7 (17.43, 54.25)**	**<.001**
	IgE^c^	**20.14**	**123.69**	**8.47 (3.52, 20.40)**	**<.001**
	IgG4^c^	12.99	12.39	1.28 (0.66, 2.51)	.454
	Total IgE^b^	675.19	744.28	1.23 (0.89, 1.70)	.201
	Total IgG4^c^	20,461.92	14,578.97	0.55 (0.28, 1.08)	.082

Significant associations (*p* ≤ .05) are highlighted in bold.

Abbreviations: aGMR, adjusted geometric mean ratio; 95% CI, 95% confidence interval; SEA, *Schistosoma* egg antigen; Sm+, positive Kato‐Katz and/or PCR test for diagnosis of current infection with *S*.* mansoni*; Sm−, negative Kato‐Katz and PCR test for diagnosis of current infection with *S*.* mansoni*; SWA, *Schistosoma* adult worm antigen.

^a^ Reference category,

^b^ Antibody levels detected by ImmunoCAP®. Concentrations are in kU/L.

^c^ Antibody levels detected by ELISA. Concentrations are in ng/ml.

^d^ All geometric mean ratios and 95% confidence intervals adjusted for survey design, age and sex.

^e^ Geometric mean ratios and 95% confidence intervals for associations between antibody levels and SPT reactivity were additionally adjusted for *Sm* result.

In the urban survey (Table 3), *Sm* infection was positively associated with total and SWA‐ and SEA‐specific IgE and IgG4 (*p* < .001), and ELISA‐determined HDM‐specific IgE (*p* = .037), cockroach‐specific IgE (*p* = .040) and cockroach‐specific IgG4 concentrations (*p* = .005). Cockroach SPT reactivity was positively associated with cockroach‐specific IgE (ImmunoCAP^®^: 13.49 [7.19, 25.32], *p* < .001; ELISA: 4.64 [2.00, 10.78], *p* = .001), cockroach‐specific IgG4 (3.45 [1.76, 6.77], *p* = .001) and total IgE concentrations (4.19 [2.80, 6.28], *p* < .001). Despite a positive association with SEA‐specific IgE (1.13 [1.03, 1.23], *p* = .012), cockroach SPT reactivity was inversely associated with SEA‐specific IgG4 (0.48 [0.27, 0.86], *p* = .016). HDM SPT reactivity was positively associated with HDM‐specific IgE (ImmunoCAP^®^: 47.48 [24.1, 93.68], *p* < .001; ELISA: 24.69 [12.9, 47.39], *p* < .001), HDM‐specific IgG4 (2.10 [1.53, 2.87], *p* < .001) and total IgE concentrations (2.46 [1.65, 3.67], *p* < .001).

**TABLE 3:** Urban survey: associations between antibody (IgE and IgG4) concentrations and current *S*.* mansoni* infection and skin prick test reactivity

Antigen	Antibody	Geometric mean		aGMR (95% CI)^d^, ^e^	*p* value
		*Sm‐* ^a^	*Sm+*		
SWA	IgE^c^	**2188**	**3949**	**1.75 (1.58, 1.94)**	**<.001**
	IgG4^c^	**12,134**	**80,715**	**5.93 (3.76, 9.38)**	**<.001**
SEA	IgE^c^	**2434**	**4660**	**1.88 (1.63, 2.16)**	**<.001**
	IgG4^c^	**981**	**83,098**	**65.9 (41.4, 104.8)**	**<.001**
House dust mite	IgE^b^	0.18	0.25	1.39 (0.65, 3.00)	.379
	IgE^c^	**16.57**	**38.20**	**2.08 (1.05, 4.12)**	**.037**
	IgG4^c^	19.56	29.02	1.62 (0.99, 2.66)	.056
German cockroach	IgE^b^	0.18	0.22	1.09 (0.63, 1.88)	.745
	IgE^c^	**18.04**	**38.12**	**1.91 (1.03, 3.53)**	**.040**
	IgG4^c^	**8.78**	**24.28**	**2.54 (1.37, 4.71)**	**.005**
	Total IgE^b^	**143.21**	**360.22**	**2.62 (1.81, 3.79)**	**<.001**
	Total IgG4^c^	**12,196.94**	**25,104.18**	**1.96 (1.45, 2.64)**	**<.001**

		*Cockroach SPT‐* ^a^	*Cockroach SPT+*		
SWA	IgE^c^	2367	2551	1.08 (0.99, 1.16)	.057
	IgG4^c^	15,174	21,505	1.41 (0.87, 2.30)	.152
SEA	IgE^c^	**2645**	**3187**	**1.13 (1.03, 1.23)**	**.012**
	IgG4^c^	**2131**	**1171**	**0.28 (0.12, 0.63)**	**.004**
German cockroach	IgE^b^	**0.13**	**1.70**	**13.49 (7.19, 25.32)**	**<.001**
	IgE^c^	**16.39**	**79.46**	**4.64 (2.00, 10.78)**	**.001**
	IgG4^c^	**9.08**	**23.59**	**3.45 (1.76, 6.77)**	**.001**
	Total IgE^b^	**140.72**	**505.65**	**4.19 (2.80, 6.28)**	**<.001**
	Total IgG4^c^	12,517.24	17,417.20	1.32 (0.83, 2.12)	.223

		*Dust mite* *SPT−* ^a^	*Dust mite SPT+*		
SWA	IgE^c^	2365	2526	0.99 (0.91, 1.09)	.915
	IgG4^c^	15,601	19,133	0.88 (0.44, 1.75)	.707
SEA	IgE^c^	2655	3018	1.02 (0.86, 1.19)	.835
	IgG4^c^	1884	2337	0.55 (0.26, 1.17)	.113
House dust mite	IgE^b^	**0.09**	**4.86**	**47.48 (24.1, 93.68)**	**<.001**
	IgE^c^	**10.34**	**297.85**	**24.69 (12.9, 47.39)**	**<.001**
	IgG4^c^	**20.05**	**32.33**	**2.10 (1.53, 2.87)**	**<.001**
	Total IgE^b^	**141.85**	**346.98**	**2.46 (1.65, 3.67)**	**<.001**
	Total IgG4^c^	12,526.67	16,210.02	1.26 (0.85, 1.87)	.233

Significant associations (*p* ≤ .05) are highlighted in bold.

Abbreviations: aGMR, adjusted geometric mean ratio; 95% CI, 95% confidence interval; SEA, *Schistosoma* egg antigen; Sm+, positive Kato‐Katz and/or PCR test for diagnosis of current infection with *S*.* mansoni*; Sm−, negative Kato‐Katz and PCR test for diagnosis of current infection with *S*.* mansoni*; SWA, *Schistosoma* adult worm antigen.

^a^ reference category

^b^ Antibody levels detected by ImmunoCAP®. Concentrations are in kU/L.

^c^ Antibody levels detected by ELISA. Concentrations are in ng/ml.

^d^ All geometric mean ratios and 95% confidence intervals adjusted for survey design, age and sex.

^e^ Geometric mean ratios and 95% confidence intervals for associations between antibody levels and SPT reactivity were additionally adjusted for *Sm* result.

Akin to observations in the rural and urban surveys, cockroach and HDM SPT reactivity were generally positively associated with total and with cockroach‐ and HDM‐specific IgE concentrations, respectively, among both asthma cases and non‐asthmatic controls (Table S2).

### 3.3 Associations between antibody ratios and current *S. mansoni* infection and skin prick test reactivity

**FIGURE 2:**
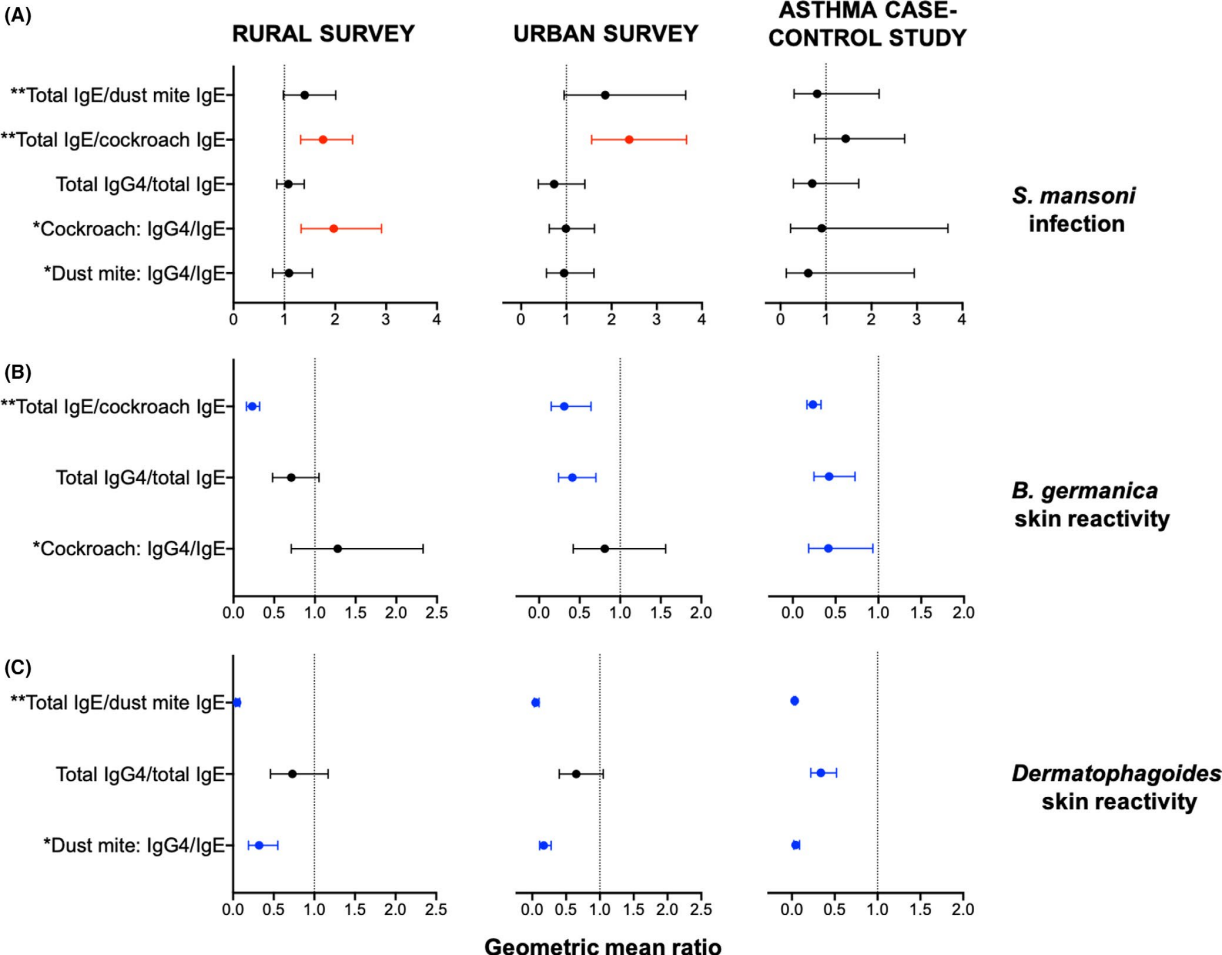
Associations between antibody ratios and *Schistosoma mansoni* infection and skin prick test reactivity. Forest plots show geometric mean ratios (GMRs) and 95% confidence intervals (95% CIs) for associations between antibody ratios and (A) current *S*.* mansoni* infection and (B, C) allergen skin prick test reactivity. Raw antibody responses were skewed, so log_10_‐transformed antibody data were used in our linear regression models; we back‐transformed the results to obtain GMRs and 95% CIs. All GMRs and 95% CIs were adjusted for age and sex, and additionally for survey design in the rural and urban surveys. GMRs and 95% CIs for associations with skin prick test reactivity were additionally adjusted for *S*.* mansoni* infection status. Red colour denotes associations where GMR and 95% CI >1; blue colour denotes associations where GMR and 95% CI <1, and black colour denotes lack of a significant association. ^*^Antibody levels detected by ELISA. Concentrations are in ng/ml. ^**^Antibody levels detected by ImmunoCAP®. Concentrations are in kU/L

*Schistosoma mansoni* infection was positively associated with total IgE/cockroach‐specific IgE ratios in both community surveys (rural: 1.76 [1.31, 2.34], *p* < .001; urban: 2.39 [1.56, 3.66], *p* < .001), and with cockroach‐specific IgG4/IgE ratios in the rural survey (1.97 [1.33, 2.91], *p* = .002) [Figure 2A]. There were no significant associations between *Sm* infection and antibody ratios in the asthma case‐control study.

In all three studies, cockroach SPT reactivity was inversely associated with total IgE/cockroach‐specific IgE ratios (rural: 0.23 [0.16, 0.32], *p* < .001; urban: 0.31 [0.15, 0.64], *p* = .003; asthma study: 0.23 [0.17, 0.33], *p* < .001) [Figure 2B]. Cockroach SPT reactivity was also inversely associated with total IgG4/total IgE ratios in the urban survey (0.41 [0.24, 0.70], *p* = .002) and in the asthma case‐control study (0.42 [0.25, 0.73], *p* = .002), and with cockroach‐specific IgG4/IgE ratios in the asthma case‐control study (0.42 [0.19, 0.94], *p* = .034) [Figure 2B].

In all three studies, HDM SPT reactivity was inversely associated with total IgE/HDM‐specific IgE ratios (rural: 0.05 [0.03, 0.08], *p* < .001; urban: 0.05 [0.03, 0.10], *p* < .001; asthma study: 0.03 [0.02, 0.04], *p* < .001), and HDM‐specific IgG4/IgE ratios (rural: 0.32 [0.19, 0.55], *p* < .001; urban: 0.17 [0.11, 0.28], *p* < .001; asthma study: 0.04 [0.02, 0.09], *p* < .001) [Figure 2C]. HDM SPT reactivity was also inversely associated with total IgG4/total IgE ratios in the asthma case‐control study (0.34 [0.22, 0.52], *p* < .001) [Figure 2C].

Assessment of associations between antibody ratios and skin prick test reactivity, independently among asthma cases and non‐asthmatic controls, showed observations akin to the above: cockroach and HDM SPT reactivity were inversely associated with allergen‐specific IgG4/IgE ratios, total IgE/allergen‐specific IgE ratios and total IgG4/total IgE ratios (Table S3).

### 3.4 Rural‐urban comparison of plasma IgE and IgG4 levels

Urban participants, compared with rural survey participants, had lower geometric mean concentrations of SEA‐ and SWA‐specific IgE, IgG and IgG4 (*p* < .001), cockroach‐specific IgE (ImmunoCAP^®^: *p* = .041; ELISA: *p* = 0.017) and IgG4 (*p* = .038), total IgE (*p* < .001), total IgG4 (*p* = .011), total IgE/cockroach‐specific IgE ratios (*p* < .001) and total IgE/HDM‐specific IgE ratios (*p* < .001) [Table 4]. However, HDM‐specific IgE (ImmunoCAP^®^, *p* = .038) and IgG4 (*p* = .001), and HDM‐specific IgG4/IgE ratios (*p* = .011), were higher in the urban survey.

**TABLE 4:** Rural‐urban comparison of IgE and IgG4 levels

Antigen	Antibody / antibody ratio	Geometric mean		aGMR (95% CI)^d^	*p* value
		*Rural* ^a^	*Urban*		
SWA	IgE^c^	**4452.34**	**2386.55**	**0.56 (0.52, 0.61)**	**<.001**
	IgG4^c^	**82,045.65**	**16,091.34**	**0.24 (0.16, 0.35)**	**<.001**
SEA	IgE^c^	**4340.73**	**2714.94**	**0.63 (0.56, 0.71)**	**<.001**
	IgG4^c^	**90,796.70**	**1957.08**	**0.03 (0.02, 0.06)**	**<.001**
German cockroach	IgE^b^	**0.31**	**0.19**	**0.71 (0.50, 0.99)**	**.041**
	IgE^c^	**35.81**	**20.65**	**0.62 (0.42, 0.91)**	**.017**
	IgG4^c^	**13.97**	**10.31**	**0.74 (0.56, 0.98)**	**.038**
	IgG4/IgE ratio^c^	5.40	5.58	1.00 (0.70, 1.44)	.984
House dust mite	IgE^b^	**0.14**	**0.19**	**1.63 (1.03, 2.60)**	**.038**
	IgE^c^	24.49	18.91	0.86 (0.60, 1.24)	.419
	IgG4^c^	**12.89**	**21.91**	**1.54 (1.20, 1.96)**	**.001**
	IgG4/IgE ratio^c^	**11.86**	**21.44**	**1.58 (1.12, 2.24)**	**.011**
	Total IgE^b^	**682.57**	**166.23**	**0.29 (0.22, 0.39)**	**<.001**
	Total IgG4^c^	**19,689.40**	**13,254.08**	**0.71 (0.55, 0.92)**	**.011**
	Total IgG4/total IgE ratio	**37.58**	**98.93**	**2.54 (1.98, 3.27)**	**<.001**
	Total IgE/cockroach IgE ratio^b^	**2025.34**	**895.46**	**0.45 (0.34, 0.60)**	**<.001**
	Total IgE/dust mite IgE ratio^b^	**3948.87**	**898.91**	**0.23 (0.16, 0.33)**	**<.001**

Significant associations (*p* ≤ .05) are highlighted in bold.

Abbreviations: aGMR, adjusted geometric mean ratio; 95% CI, 95% confidence interval; SEA, *Schistosoma* egg antigen; SWA, *Schistosoma* adult worm antigen.

^a^ Reference category.

^b^ Antibody levels detected by ImmunoCAP®. Concentrations are in kU/L.

^c^ Antibody levels detected by ELISA. Concentrations are in ng/ml.

^d^ All geometric mean ratios and 95% confidence intervals adjusted for survey design, age and sex.

### 3.5 Associations between antibody profiles and asthma

**FIGURE 3:**
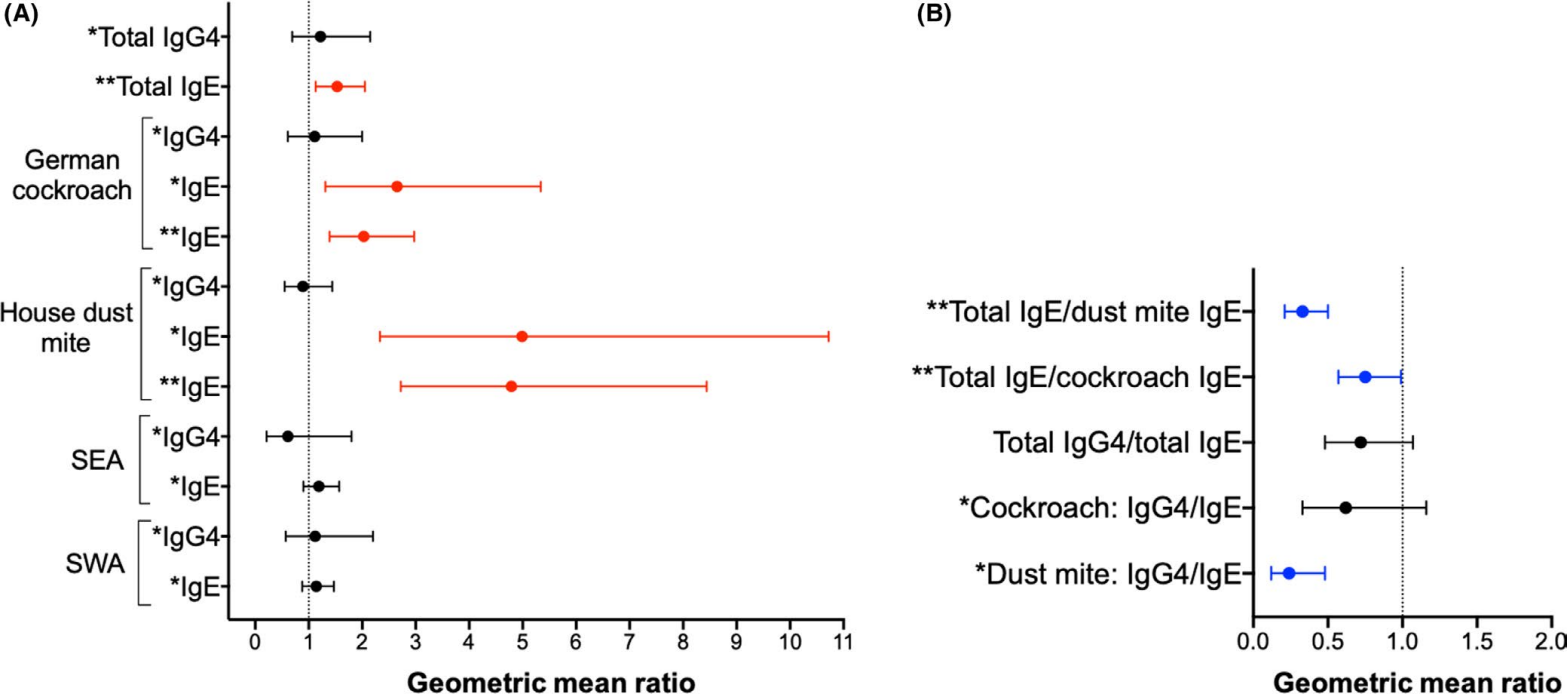
Asthma case‐control study: associations between asthma and antibody concentrations and ratios. Forest plots show geometric mean ratios (GMRs) and 95% confidence intervals (95% CIs) for associations between asthma and antibody concentrations (A) and ratios (B). Raw antibody responses were skewed, so log_10_‐transformed antibody data were used in our linear regression models; we back‐transformed the results to obtain GMRs and 95% CIs. All GMRs and 95% CIs were adjusted for age and sex. Red colour denotes associations where GMR and 95% CI >1; blue colour denotes associations where GMR and 95% CI <1, and black colour denotes lack of a significant association. ^*^Antibody levels detected by ELISA. Concentrations are in ng/ml. ^**^Antibody levels detected by ImmunoCAP®. Concentrations are in kU/L. SEA, *Schistosoma* egg antigen; SWA, *Schistosoma* adult worm antigen

Asthma was positively associated with total IgE (1.53 [1.13, 2.05], *p* = .005), cockroach‐specific IgE (ELISA: 2.65 [1.31, 5.34], *p* = .007; ImmunoCAP^®^: 2.03 [1.39, 2.97], *p* < .001) and HDM‐specific IgE (ELISA: 4.99 [2.33, 10.72], *p* < .001; ImmunoCAP^®^: 4.79 [2.72, 8.44], *p* < .001) [Figure 3A]. Conversely, there were inverse associations between asthma and total IgE/HDM‐specific IgE ratios (0.33 [0.21, 0.50], *p* < .001), total IgE/cockroach‐specific IgE ratios (0.75 [0.57, 0.99], *p* = .047) and HDM‐specific IgG4/IgE ratios (0.24 [0.12, 0.48], *p* < 0.001) [Figure 3B].

Asthma is a syndrome of different phenotypes^38^ and is sometimes broadly grouped into “allergic” and “non‐allergic” phenotypes based on atopic sensitization. In our case‐control study, “allergic” (SPT positive) asthma cases had lower HDM‐specific IgG4/IgE, total IgG4/total IgE and total IgE/allergen‐specific IgE ratios, compared with “non‐allergic” (SPT negative) asthma cases (Table S3 and Figure S1A and S1B **)**. Furthermore, HDM SPT–positive asthma cases had lower HDM‐specific IgG4/IgE, total IgG4/total IgE and total IgE/HDM‐specific IgE ratios, compared with both SPT‐negative and SPT‐positive controls (Table S3 and Figure S1A **)**. The same trend was observed for comparisons between cockroach SPT‐positive asthma cases and both SPT‐negative and SPT‐positive controls, albeit without statistical significance (Figure S1B). Interestingly, HDM and cockroach SPT‐negative asthma cases had lower geometric means of allergen‐specific IgG4/IgE and total IgE/allergen‐specific IgE ratios than SPT‐negative controls, but higher levels compared with SPT‐positive controls (Table S3).

## 4 DISCUSSION

We assessed total, *Schistosoma*‐ and allergen‐specific IgE and IgG4 concentrations among participants of three large population‐based studies in Uganda and found strong inverse associations between allergen SPT reactivity and *Schistosoma*‐specific IgG4, allergen‐specific IgG4/IgE ratios and total IgE/allergen‐specific IgE ratios. Importantly, doctor‐diagnosed asthma cases also had significantly lower allergen‐specific IgG4/IgE ratios and total IgE/allergen‐specific IgE ratios compared with non‐asthmatic controls. *Sm* infection and the rural (vs. urban) environment were positively associated with total, *Schistosoma*‐ and allergen‐specific IgE and IgG4, and with allergen‐specific IgG4/IgE ratios and total IgE/allergen‐specific IgE ratios.

Host responses to helminths exhibit significant similarities with responses to common allergens, owing to related molecular targets.^39^, ^40^, ^41^, ^42^ This probably explains observed positive associations between *Sm* infection and allergen‐specific IgG4 and IgE among participants of our rural and urban community surveys. Strong positive associations between *Sm* infection and total IgE are consistent with observations of helminth‐induced polyclonal stimulation of non‐specific IgE.^18^ It is plausible that helminths potentiate synthesis of this seemingly superfluous IgE primarily as an immune evasion mechanism: disproportionately elevated levels of non–helminth‐specific IgE saturate available IgE receptors (FcεRIs) on effector cells, inhibiting specific mediator release.^43^ This is expected to have benefits for the helminth, but also inadvertent bystander effects on allergic responses, as allergens will be outcompeted and less likely to cross‐link FcεRI‐bound IgE.^14^, ^20^, ^21^

Mitre et al.^44^ initially found that a high total IgE/allergen‐specific IgE ratio did not inhibit basophil degranulation, but further experiments suggested that inhibition could occur at ratios exceeding 500:1. In the current analysis, we show that total IgE/allergen‐specific IgE ratios were strongly inversely associated with allergen SPT reactivity (irrespective of study setting), and with asthma, providing strong support for the above hypotheses. Moreover, geometric means of total IgE/allergen‐specific IgE ratios exceeded 700 among non‐asthmatic controls in the case‐control study, and 1000 among our *Sm*‐infected and SPT‐negative participants irrespective of study setting—in excess of Mitre's threshold for inhibition.

Our observations of inverse associations between allergen‐specific IgG4/IgE ratios and SPT reactivity and asthma strengthen the argument that the IgG4‐IgE balance is also important in protection against allergies. In addition, SWA‐ and SEA‐specific IgG4 concentrations were significantly lower among HDM SPT‐reactive rural survey participants and cockroach SPT‐reactive urban survey participants, respectively. Although IgG4 has been implicated in IgG4‐related disease,^45^ it appears to have a more prominent role in immune regulation^16^ and tolerance.^46^ Besides, IgG4 does not activate the complement system or lead to formation of immune complexes, and is not associated with mast cell or basophil degranulation.^47^ Its major mode of action against allergic inflammation seems to be blockage of allergen recognition by IgE, because both antibodies have similar antigenic specificity.^16^, ^48^, ^49^ Furthermore, concurrent binding of the FcεRI and the inhibitory IgG receptor (FcγRIIB) by IgE and IgG4, respectively, may result in a FcγRIIB‐dependent inhibition of IgE‐mediated effector cell activation.^49^, ^50^, ^51^ It is worth noting that other IgG classes have also been associated with IgE blocking; however, IgG4 seems to play the bigger role.^52^

There is a counter‐argument that IgG4 might not have a direct mechanistic role in protection against allergy‐related outcomes. Instead, the inverse association between IgG4 levels and IgE effector function may represent an ‘epiphenomenon’, merely reflective of parallel phenomena, such as abundance of IL‐10 (and/or TGF‐β)–producing T regulatory and B regulatory cells.^53^ However, the latter hypothesis remains to be confirmed by experimental studies.

We measured allergen extract–specific IgE using the standard ImmunoCAP^®^ test, and later, using an in‐house ELISA assay. The latter technique was used to facilitate determination of IgG4/IgE ratios, since, for logistical reasons, we could not measure allergen‐specific IgG4 by ImmunoCAP^®^. However, patterns of associations with ImmunoCAP^®^‐ and ELISA‐determined IgE were comparable (except for cockroach‐specific IgE in a few instances). We did not formally adjust for multiple statistical testing. However, our interpretations rely on patterns and magnitude of associations, and consistency and biological credibility of our findings based on other published works. It is noteworthy that our studies were cross‐sectional; therefore, it was not possible to demonstrate causal relationships between antibody profiles and *Sm* infection, the rural‐urban environment, allergic sensitization and asthma.

In conclusion, we underline the potential role of the total IgE‐allergen‐specific IgE balance and the IgG4‐IgE balance in mitigation of allergic responses, providing correlative evidence from three cross‐sectional population‐based studies. These results build upon previous findings that apportion high IgG4/IgE ratios an important role in allergen‐specific immunotherapy.^46^ Further studies in animal models would advance understanding of a potential role of IgG4 (and polyclonally stimulated IgE) in mitigation of allergy‐related disease; however, they are hindered by the lack of IgG4 in mice. Therefore, future work should focus on *in vitro* experiments using human samples. For example, a microarray screening platform could be utilized to identify helminth antigens that provoke robust serum IgG4 and little or no IgE response. Such antigens may be valuable for therapeutic approaches against allergic conditions.

## CONFLICT OF INTERESTS

The authors declare no conflict of interest.

## AUTHOR CONTRIBUTIONS

AME, GN, HM and ELW contributed to the conception and experimental design of the studies. GN, JN and JK performed the laboratory experiments. AME, HM, RES and MN led and participated in field and clinic procedures. GN analysed the results with significant input from ELW. GN wrote the manuscript, with all authors contributing to the interpretation of the results, and revision and approval of the final manuscript.

## Supporting information

Appendix S1

## Data Availability

Data are available on request via https://doi.org/10.17037/DATA.00001804. To obtain access to the data, complete the application process on the website. Requests will be reviewed and assessed by the corresponding author, in consultation with the LSHTM’s Research Data Manager and relevant LSHTM staff members responsible for research governance and data protection. Applications will be evaluated on the basis of their compatibility with the study's research objectives and the ability to provide de‐identified data sufficient to meet the intended purpose, without breaching participant confidentiality or the study's ethical and legal commitments. Successful applicants will be asked to sign a Data Transfer Agreement prior to being provided with the data.
