# Supplementary material for: Allergen skin test reactivity and asthma are inversely associated with ratios of IgG4/IgE and total IgE/allergen‐specific IgE in Ugandan communities
Source: Clin Exp Allergy. 2021 Feb 5;51(5):703–15. doi: 10.1111/cea.13834 (PMC7610822; doi:10.1111/cea.13834)
Supplement: Supplementary file 1 — Appendix S1 [file CEA-51-703-s001.docx]

**SUPPLEMENTARY INFORMATION FOR**

**Allergen skin test reactivity and asthma are inversely associated with ratios of IgG4/IgE and total IgE/allergen-specific IgE in Ugandan communities**

*Gyaviira Nkurunungi, Jacent Nassuuna, Harriet Mpairwe, Joyce Kabagenyi, Margaret Nampijja, Richard E Sanya, Emily L Webb, Alison M Elliott*

**Supplementary methods**

*Schistosoma adult worm (SWA)- and egg (SEA)-specific IgE and IgG4 ELISA*

All but the first 2 columns of 4HBX Immulon (Thermo Scientific, NY, USA) 96-well plates were coated with 50μl of SWA [8 μg/ml] or SEA [2.4 μg/ml] (purchased from Professor Michael J Doenhoff, University of Nottingham) in bicarbonate (Na_2_CO_3_ + NaHCO_3_) buffer (0.1M, pH 9.6). Two-fold dilutions of human IgE (Calbiochem, Beeston, UK) or IgG4 (Sigma-Aldrich) standard, diluted in bicarbonate buffer, were added to the first 2 columns of each plate to form standard curves. The plates were then incubated overnight at 4ºC. Plates were washed with phosphate-buffered saline (PBS 1X)-tween 20 solution, blocked with 150μl of 1% skimmed milk diluted in PBS-Tween 20 at room temperature (RT), and incubated overnight at 4ºC with 50μl of plasma samples diluted 1/20 (IgE assay) or 1/200 (IgG4 assay) with 0.1% skimmed milk in PBS-Tween 20 (assay buffer). Plates were washed and antibody binding detected by incubating the plates overnight at 4ºC with 0.5μg/ml of biotinylated monoclonal mouse anti-human IgE or IgG4 (BD Pharmingen™). This was followed by a 1-hour incubation with a streptavidin-Horseradish Peroxidase (strep-HRP) conjugate (Mast Group Ltd, Bootle, UK), diluted 1/4000 with assay buffer, at RT. Plates were developed by addition of 100μl of o-phenylenediamine (Sigma-Aldrich) and reactions stopped after 30 minutes with 25μl of 2M Sulphuric acid. Optical density values were measured at 490nm (reference wavelength 630nm) on a 96-well plate ELISA reader. IgE or IgG4 concentrations (ng/ml) were interpolated from standard curves using a five-parameter curve fit using Gen5 data collection and analysis software (BioTek Instruments Inc, Vermont, Winooski, USA).

### *Measurement of allergen-specific IgG4 levels by ELISA*

All but the first 2 columns of MICROLON^®^ high binding 96-well plates (Greiner bio-one, UK) were coated with 50μl of *D. pteronyssinus* or *B. germanica* allergen extracts (Greer Labs, Lenoir, NC, USA) at a concentration of 5μg/ml in bicarbonate (Na_2_CO_3_ + NaHCO_3_) buffer (0.1M, pH 9.6). Two-fold dilutions of human IgG4 (Sigma-Aldrich) standard, diluted in bicarbonate buffer, were added to the first 2 columns of each plate to form standard curves. The plates were then incubated overnight at 4ºC. Plates were washed with phosphate-buffered saline (PBS)-tween 20 solution, blocked with 150μl of skimmed milk diluted in PBS-Tween 20 at room temperature (RT), and incubated overnight at 4ºC with 50μl of plasma samples diluted 1/40 with 10% fetal bovine serum in PBS-Tween 20 (assay buffer). Plates were washed and antibody binding detected by incubating the plates overnight at 4ºC with 0.5μg/ml of biotinylated monoclonal mouse anti-human IgG4 (BD Pharmingen™), followed by a one-hour incubation with a streptavidin-Horseradish Peroxidase (strep-HRP) conjugate (Mast Group Ltd, Bootle, UK), diluted 1/3000 with assay buffer, at RT. Plates were developed by addition of 100μl of o-phenylenediamine (Sigma-Aldrich) and reactions stopped with 30μl of 2M Sulphuric acid. Optical density values were measured at 490nm (reference wavelength 630nm) on a 96-well plate ELISA reader. IgG4 concentrations (ng/ml) were interpolated from standard curves using a five-parameter curve fit using Gen5 data collection and analysis software (BioTek Instruments Inc, Vermont, Winooski, USA).

### *Measurement of total IgG4 levels by ELISA*

### 96-well Maxisorp plates (VWR, U.S.A) were coated with purified mouse anti-human IgG4 (BD Pharmingen™) in bicarbonate buffer (0.1 M, pH 9.6) overnight at 4ºC, and blocked for one hour with 3% skimmed milk in 1X PBS at room temperature (RT). Plates were then incubated with plasma samples (diluted 1/800 in 0.1M Tris pH 7.5 + 0.05% Tween 20) and IgG4 standards (Sigma Aldrich) for one hour. Antibody binding was detected by incubating the plates with mouse anti-human IgG4 conjugated to horseradish peroxidase (Invitrogen) for one hour, followed by a colour reaction with o-phenylenediamine (Sigma Aldrich). Reactions were stopped with 2M Sulphuric acid. Absorbance was measured at 490nm (reference wavelength 630nm) on a 96-well plate ELISA reader. IgG4 concentrations (ng/ml) were interpolated from standard curves using a five-parameter curve fit using Gen5 data collection and analysis software (BioTek Instruments Inc, Vermont, Winooski, USA).

### *Measurement of total IgE and allergen-specific IgE using the ImmunoCAP® test*

The ImmunoCAP® assay uses an automated system that loads serum / plasma on **1)** a hydrophilic polymer sponge (the ‘ImmunoCAP’) covalently coupled to an allergen (for specific IgE measurement) or **2)** an ImmunoCAP reaction vessel covalently coupled to an anti-IgE antibody (which reacts with total IgE in sample). Unbound IgE is washed off and a conjugate (anti-IgE antibody grafted with β-galactosidase) is added. The supernatant is then aspirated, and a substrate (methyl-umbelliferyl-β-D galactoside) deposited on the ImmunoCAP sponge. A stop solution is added and the sponge then compressed. The fluorescence generated by the resultant eluate, a product of enzyme degradation (methyl umbelliferone, proportional to concentration of IgE in serum/plasma), is measured. Results are reported quantitatively using a kU/L scale. The calibrator is IgE bound to anti-IgE caps using a six-point quantitative curve. Calibration concentration ranges from 0 to 100 kU/L for specific IgE and 2 to 5000 kU/L for total IgE. A cut-off of 0.35 kU/L was used to define allergen-specific IgE sensitisation, as recommended by the test manufacturer.[^1^](#_ENREF_1)

### *Measurement of allergen-specific IgE levels by ELISA*

All but the first 2 columns of MICROLON® high binding 96-well plates (Greiner bio-one, UK) were coated with 50μl of *D. pteronyssinus* or *B. germanica* allergen extracts (Greer Labs, Lenoir, NC, USA) at a concentration of 5μg/ml in bicarbonate (Na_2_CO_3_ + NaHCO_3_) buffer (0.1M, pH 9.6). Two-fold dilutions of human IgE (Calbiochem, Beeston, UK) standard, diluted in bicarbonate buffer, were added to the first 2 columns of each plate to form standard curves. The plates were then incubated overnight at 4ºC. Plates were washed with phosphate-buffered saline (PBS)-Tween 20 solution, blocked with 150μl of skimmed milk diluted in PBS-Tween 20 at room temperature (RT), and incubated overnight at 4ºC with 50μl of plasma samples diluted 1/20 with 10% fetal bovine serum in PBS-Tween 20 (assay buffer). Plates were washed and antibody binding detected by incubating the plates overnight at 4ºC with 0.5μg/ml of biotinylated monoclonal mouse anti-human IgE (BD Pharmingen™), followed by a one-hour incubation with a streptavidin-Horseradish Peroxidase (strep-HRP) conjugate (Mast Group Ltd, Bootle, UK), diluted 1/3000 with assay buffer, at RT. Plates were developed by addition of 100μl of o-phenylenediamine (Sigma-Aldrich) and reactions stopped with 30μl of 2M Sulphuric acid. Optical density values were measured at 490nm (reference wavelength 630nm) on a 96-well plate ELISA reader. IgE concentrations (ng/ml) were interpolated from standard curves using a five-parameter curve fit using Gen5 data collection and analysis software (BioTek Instruments Inc, Vermont, Winooski, USA).

**Supplementary results**

**Table S1.** Impact of community-based intensive versus standard anthelminthic treatment on IgE and IgG4 profiles in the rural survey

| **Outcome** | Geometric mean | |  | Unadjusted | |  | Adjusted for age & sex | |
| --- | --- | --- | --- | --- | --- | --- | --- | --- |
|  | **Standard*** | **Intensive** |  | **GMR** **(95% CI)** | **p** |  | **GMR** **(95% CI)** | **p** |
| ***Schistosoma-*specific antibody concentration (ELISA, ng/ml)** |  |  |  |  |  |  |  |  |
| SWA-specific IgE | 4447 | 4440 |  | 0.99 (0.88, 1.14) | 0.98 |  | 0.99 (0.87, 1.14) | 0.95 |
| SWA-specific IgG4 | 90851 | 71491 |  | 0.79 (0.50, 1.23) | 0.28 |  | 0.78 (0.52, 1.17) | 0.22 |
| SEA-specific IgE | 4191 | 4463 |  | 1.07 (0.90, 1.26) | 0.45 |  | 1.06 (0.90, 1.25) | 0.44 |
| SEA-specific IgG4 | 108695 | 69108 |  | 0.64 (0.29, 1.41) | 0.25 |  | 0.65 (0.29, 1.45) | 0.28 |
| **asIgE concentration (ImmunoCAP, kU/L)^#^** |  |  |  |  |  |  |  |  |
| House dust mite (*D. pteronyssinus*) | 0.16 | 0.13 |  | 0.78 (0.51, 1.17) | 0.22 |  | 0.76 (0.51, 1.13) | 0.17 |
| German cockroach (*B. germanica*) | 0.35 | 0.29 |  | 0.82 (0.55, 1.22) | 0.31 |  | 0.81 (0.55, 1.20) | 0.28 |
| **asIgE and asIgG4 concentration (ELISA, ng/ml)** |  |  |  |  |  |  |  |  |
| House dust mite-specific IgE | 27.7 | 22.7 |  | 0.82 (0.48, 1.39) | 0.45 |  | 0.81 (0.49, 1.35) | 0.40 |
| House dust mite-specific IgG4 | 14.0 | 12.0 |  | 0.86 (0.63, 1.17) | 0.32 |  | 0.89 (0.68, 1.2) | 0.37 |
| Cockroach-specific IgE | 33.6 | 40.3 |  | 1.20 (0.76, 1.89) | 0.41 |  | 1.28 (0.81, 2.01) | 0.28 |
| Cockroach-specific IgG4 | 14.9 | 13.6 |  | 0.91 (0.57, 1.47) | 0.69 |  | 0.93 (0.59, 1.46) | 0.75 |
| **asIgG4/asIgE ratios** |  |  |  |  |  |  |  |  |
| House dust mite-specific IgG4/IgE ratio | 11.5 | 11.9 |  | 1.04 (0.63, 1.70) | 0.88 |  | 1.05 (0.66, 1.64) | 0.84 |
| Cockroach-specific IgG4/IgE ratio | 6.3 | 4.7 |  | 0.74 (0.48, 1.16) | 0.18 |  | 0.71 (0.47, 1.08) | 0.10 |
|  |  |  |  |  |  |  |  |  |
| **Total IgE** (ImmunoCAP, kU/L) | 761 | 606 |  | 0.80 (0.61, 1.04) | 0.09 |  | 0.79 (0.62, 1.00) | 0.05 |
| **Total IgG4** (ELISA, ng/ml) | 22117 | 17176 |  | 0.78 (0.55, 1.10) | 0.15 |  | 0.78 (0.55, 1.08) | 0.13 |
| **Total IgG4/total IgE ratio** (ELISA/ImmunoCAP) | 36.4 | 38.8 |  | 1.07 (0.88, 1.30) | 0.50 |  | 1.05 (0.87, 1.26) | 0.60 |
| **Total IgE/cockroach IgE ratio** (ImmunoCAP) | 2089 | 1963 |  | 0.94 (0.71, 1.25) | 0.66 |  | 0.94 (0.71, 1.25) | 0.65 |
| **Total IgE/dust mite IgE ratio** (ImmunoCAP) | 3979 | 3952 |  | 0.99 (0.71, 1.39) | 0.97 |  | 0.98 (0.69, 1.38) | 0.92 |

*****reference category

^#^Log10(+0.001) transformation at the individual level. All other antibody data were log_10_ (+1)-transformed.

**asIgE:** whole allergen extract-specific IgE

**asIgG4:** whole allergen extract-specific IgG4

**GMR:** geometric mean ratio

**95% CI:** 95% confidence interval

**SWA:** *Schistosoma* adult worm antigen

**SEA:** *Schistosoma* egg antigen.

**Table S2.** Asthma case-control study: associations between antibody (IgE and IgG4) concentrations and skin prick test reactivity

|  |  | **NON-ASTHMATIC CONTROLS** | | | |  | **ASTHMA CASES** | | | |
| --- | --- | --- | --- | --- | --- | --- | --- | --- | --- | --- |
| **Antigen** | **Antibody** | **Geometric mean** | | **aGMR (95% CI)** ^‡^ | **p value** |  | **Geometric mean** | | **aGMR (95% CI)** ^‡^ | **p value** |
|  |  | *Cockroach SPT-** | *Cockroach SPT+* |  |  |  | *Cockroach SPT-** | *Cockroach SPT+* |  |  |
| **SWA** | IgE^§^ | **1516.97** | **2707.82** | **1.79 (1.01, 3.18)** | **0.046** |  | 1904.15 | 2121.93 | 1.17 (0.84, 1.61) | 0.357 |
|  | IgG4^§^ | 11195.08 | 28211.35 | 2.13 (0.56, 8.05) | 0.264 |  | 16550.81 | 10125.03 | 0.47 (0.13, 1.63) | 0.230 |
| **SEA** | IgE^§^ | 2049.64 | 1916.69 | 0.94 (0.49, 1.81) | 0.845 |  | 2793.16 | 1824.74 | 0.70 (0.47, 1.03) | 0.073 |
|  | IgG4^§^ | 2505.54 | 1349.13 | 0.46 (0.06, 3.45) | 0.445 |  | 2142.60 | 464.34 | 0.25 (0.04, 1.69) | 0.154 |
| **German cockroach** | IgE^¶^ | **0.22** | **2.11** | **9.34 (5.13, 16.99)** | **<0.001** |  | **0.36** | **3.97** | **10.68 (5.57, 20.48)** | **<0.001** |
|  | IgE^§^ | **12.13** | **157.04** | **12.30 (3.42, 44.16)** | **<0.001** |  | 39.49 | 122.35 | 2.28 (0.63, 8.32) | 0.209 |
|  | IgG4^§^ | 16.37 | 48.33 | 2.43 (0.75, 7.84) | 0.136 |  | **15.10** | **65.29** | **3.51 (1.24, 9.96)** | **0.019** |
|  |  |  |  |  |  |  |  |  |  |  |
|  | Total IgE^¶^ | **214.64** | **536.82** | **2.45 (1.42, 4.24)** | **0.001** |  | **303.67** | **765.92** | **2.40 (1.40, 4.11)** | **0.002** |
|  | Total IgG4^§^ | 14128.99 | 15714.37 | 1.00 (0.33, 3.06) | 0.996 |  | 17456.55 | 14257.40 | 0.80 (0.27, 2.37) | 0.691 |
|  |  |  |  |  |  |  |  |  |  |  |
|  |  | *Dust mite SPT-** | *Dust mite SPT+* |  |  |  | *Dust mite SPT-** | *Dust mite SPT+* |  |  |
| **SWA** | IgE^§^ | 1586.29 | 1967.70 | 1.22 (0.73, 2.04) | 0.453 |  | 2021.22 | 1886.94 | 0.80 (0.61, 1.04) | 0.097 |
|  | IgG4^§^ | 11703.32 | 18394.99 | 1.23 (0.38, 4.03) | 0.731 |  | 19568.22 | 10650.25 | 0.37 (0.13, 1.06) | 0.063 |
| **SEA** | IgE^§^ | 2134.62 | 1724.76 | 0.78 (0.43, 1.40) | 0.400 |  | **2942.16** | **2112.08** | **0.67 (0.49, 0.94)** | **0.019** |
|  | IgG4^§^ | 3101.42 | 834.27 | 0.21 (0.03, 1.22) | 0.082 |  | 1567.46 | 1359.73 | 0.55 (0.11, 2.77) | 0.467 |
| **House dust mite** | IgE^¶^ | **0.12** | **7.57** | **64.90 (35.10, 119.99)** | **<0.001** |  | **0.15** | **26.01** | **140.70 (78.73, 251.46)** | **<0.001** |
|  | IgE^§^ | **6.65** | **570.37** | **79.35 (27.29, 230.72)** | **<0.001** |  | **9.39** | **1381.89** | **96.54 (37.06, 251.49)** | **<0.001** |
|  | IgG4^§^ | 60.82 | 127.98 | 2.23 (0.96, 5.22) | 0.063 |  | **34.14** | **104.00** | **2.76 (1.37, 5.56)** | **0.005** |
|  |  |  |  |  |  |  |  |  |  |  |
|  | Total IgE^¶^ | **193.43** | **559.68** | **3.10 (1.93, 4.97)** | **<0.001** |  | **197.55** | **820.10** | **3.76 (2.48, 5.70)** | **<0.001** |
|  | Total IgG4^§^ | 13091.94 | 19168.66 | 1.28 (0.46, 3.51) | 0.636 |  | 19349.38 | 14169.18 | 0.70 (0.30, 1.61) | 0.392 |

Significant associations (p≤0.05) are highlighted in bold.

*reference category

^¶^Antibody levels detected by ImmunoCAP®. Concentrations are in kU/L.

^§^Antibody levels detected by ELISA. Concentrations are in ng/ml.

^‡^All geometric mean ratios and 95% confidence intervals adjusted for age, sex and *Sm* infection status.

**aGMR:** adjusted geometric mean ratio; **95% CI:** 95% confidence interval; **SWA:** *Schistosoma* adult worm antigen; **SEA:** *Schistosoma* egg antigen.

**Table S3.** Asthma case-control study: associations between antibody ratios and skin prick test reactivity

|  | **NON-ASTHMATIC CONTROLS** | | | |  | **ASTHMA CASES** | | | |
| --- | --- | --- | --- | --- | --- | --- | --- | --- | --- |
|  | **Geometric mean** | | **aGMR (95% CI)** ^‡^ | **p value** |  | **Geometric mean** | | **aGMR (95% CI)** ^‡^ | **p value** |
|  | *Cockroach SPT-** | *Cockroach SPT+* |  |  |  | *Cockroach SPT-** | *Cockroach SPT+* |  |  |
| **Cockroach**-specific IgG4/IgE ratio^§^ | **11.75** | **2.46** | **0.20 (0.06, 0.67)** | **0.010** |  | 5.01 | 5.86 | 0.99 (0.34, 2.91) | 0.981 |
| Total IgG4/total IgE ratio | **96.67** | **47.23** | **0.44 (0.20, 0.95)** | **0.037** |  | **74.23** | **27.59** | **0.40 (0.19, 0.87)** | **0.020** |
| Total IgE/cockroach IgE ratio^¶^ | **1000.05** | **257.07** | **0.26 (0.16, 0.43)** | **<0.001** |  | **841.04** | **195.04** | **0.23 (0.14, 0.37)** | **<0.001** |
|  | *Dust mite SPT-** | *Dust mite SPT+* |  |  |  | *Dust mite SPT-** | *Dust mite SPT+* |  |  |
| **House dust mite**-specific IgG4/IgE ratio^§^ | **119.65** | **4.22** | **0.04 (0.01, 0.12)** | **<0.001** |  | **46.76** | **2.66** | **0.07 (0.03, 0.18)** | **<0.001** |
| Total IgG4/total IgE ratio | **98.60** | **53.85** | **0.45 (0.22, 0.91)** | **0.027** |  | **114.30** | **29.07** | **0.29 (0.16, 0.50)** | **<0.001** |
| Total IgE/dust mite IgE ratio^¶^ | **1610.72** | **77.30** | **0.05 (0.03, 0.08)** | **<0.001** |  | **1325.10** | **33.93** | **0.03 (0.02, 0.04)** | **<0.001** |

Significant associations (p≤0.05) are highlighted in bold.

*reference category

^¶^Antibody levels detected by ImmunoCAP®. Concentrations are in kU/L.

^§^Antibody levels detected by ELISA. Concentrations are in ng/ml.

^‡^All geometric mean ratios and 95% confidence intervals adjusted for age, sex and *Sm* infection status.

**aGMR:** adjusted geometric mean ratio; **95% CI:** 95% confidence interval.

| 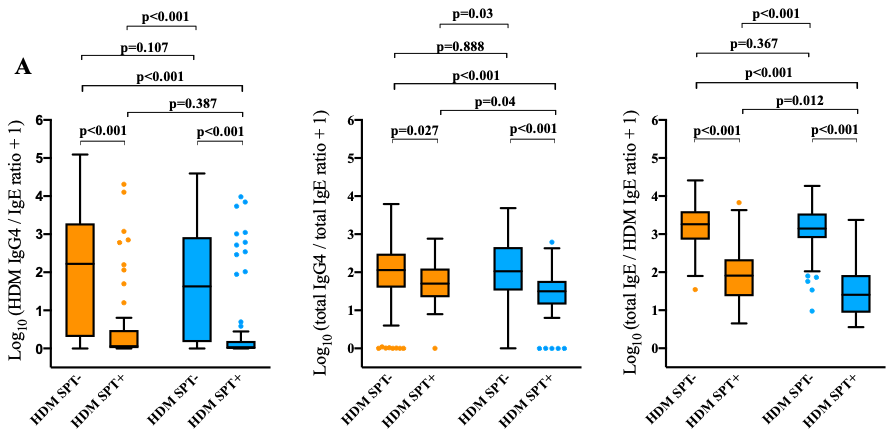 |
| --- |
| 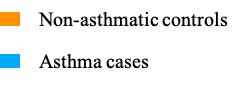 |
| 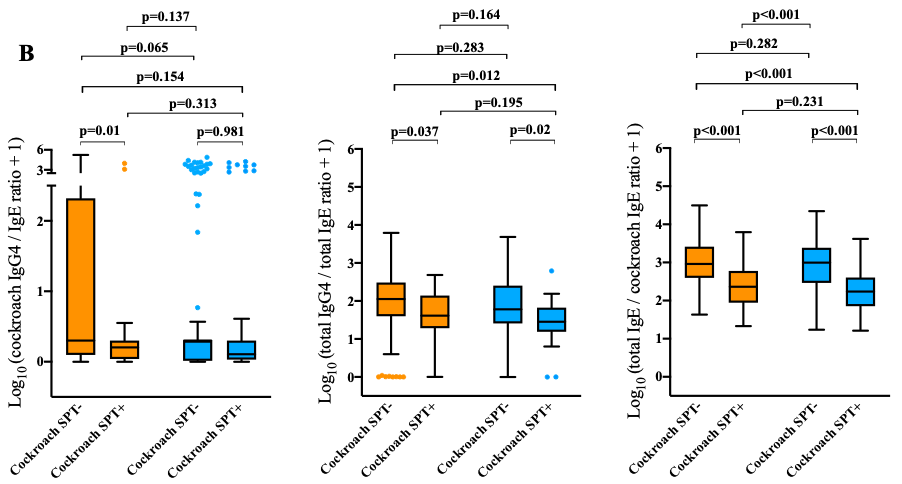 |
| **Figure S1. Asthma case-control study: associations between antibody ratios and skin prick test reactivity.** *Box-and-whisker plots show levels of antibody ratios among* ***(A)*** *house dust mite (HDM) and* ***(B)*** *German cockroach SPT positive and SPT negative asthma cases and non-asthmatic controls.*  *Horizontal lines in the plots represent medians and boxes denote interquartile ranges (IQR). Whiskers were drawn using the Tukey method (1.5 times IQR). Individual points represent outliers (>1.5 times IQR away from median).*  *Comparisons of antibody ratios between different SPT reactivity groups were conducted using linear regression analysis in Stata 15. Age-, sex- and* S. mansoni *infection-adjusted p values are shown.* |

**References**

1. ImmunoCAP Specific IgE. <http://www.phadia.com/da/Products/Allergy-testing-products/ImmunoCAP-Lab-Tests/sIgE/>. Accessed 5 July 2020.
